# 5% benzoyl peroxide is the most efficient in reducing the cutibacterium flora of the shoulder skin: a network meta-analysis

**DOI:** 10.1530/EOR-2024-0160

**Published:** 2025-06-30

**Authors:** Viktor Weninger, Gergely Agócs, Luca Hergár, Szilárd Váncsa, Bence Hegedűs, Imre Szerb, Péter Hegyi, Gábor Skaliczki

**Affiliations:** ^1^ Department of Orthopedic Surgery, Semmelweis University, Budapest, Hungary; ^2^ Centre for Translational Medicine, Semmelweis University, Budapest, Hungary; ^3^ Department of Biophysics and Radiation Biology, Semmelweis University, Budapest, Hungary; ^4^ Institute for Translational Medicine, Medical School, University of Pécs, Pécs, Hungary; ^5^ Institute of Pancreatic Diseases, Semmelweis University, Budapest, Hungary; ^6^ Uzsoki Street Hospital, Budapest, Hungary; ^7^ Department of Traumatology, Semmelweis University, Budapest, Hungary

**Keywords:** propionibacterium, benzoil, hydrogen peroxide, peroxide, infection, infection control, Cutibacterium, *C. acnes*

## Abstract

**Purpose:**

- Our study aims to compare different perioperative treatments to reduce *C. acnes*, the most common causative agent of surgical site infections following shoulder surgery.

**Methods:**

- A systematic search was performed in MEDLINE (PubMed), Embase, the Cochrane Central Register of Controlled Trials (CENTRAL), Scopus, and the Web of Science for studies published up to October 20, 2022. We included randomized-controlled trails investigating the efficacy of different dermal preparation in reducing the *C. acnes* colonising the skin surface. The studies examined positive bacterial cultures before and after skin treatment. The included trials were able to compare seven different skin treatment methods. We performed a frequentist network meta-analysis and calculated pooled risk ratios (RRs) with 95% confidence interval (CI).

**Results:**

- Our study could include comparisons of 946 different patients. The use of 5% benzoyl peroxide (BPO) and its combination with the antibiotic clindamycin was found to be the most effective in reducing *C. acnes* colonization on the skin (BPO 5% RR = 0.25, CI: 0.08–0.72, BPO with clindamycin RR = 0.25, CI: 0.04–1.50). Based on the rank plot, 5% BPO (P score: 0.808) was the most effective treatment, followed by BPO 5% with clindamycin (P score: 0.749). We could not perform a network meta-analysis regarding the efficacy of different dermal preparation in reducing *C. acnes* colonization on the dermis and in the joint.

**Conclusion:**

- Cutibacterium colonization of the skin flora can be effectively reduced on the skin surface by the use of 5% BPO.

## Introduction

Shoulder surgery is a frequently performed orthopaedic procedure (1, 2). Infection after shoulder surgery is one of the most serious complications for the patient. Patients with postoperative wound infection are more likely to spend time in intensive care and have higher morbidity rates than patients without postoperative infection (3, 4). Several attempts have been made to provide reliable, consistent, reproducible methods to decolonize or eradicate pathogens preoperatively to reduce the risk of surgical site contamination and infection (5).

*Cutibacterium acnes* (*C. acnes*), formerly *Propionibacterium acnes*, is a Gram-positive, facultatively anaerobic bacterium in the normal skin microbiome. It colonizes primarily the sebaceous glands and hair follicles of human skin and is usually non-pathogenic. However, it can also be an opportunistic pathogen, causing invasive and implant-related infections as the bacterium proliferates. The bacterium can form a biofilm, allowing it to adhere to metal implants. *C. acnes* is found in increased concentrations in the axillae, which can predispose to infection following shoulder surgery (6, 7). Several reviews have demonstrated that *C. acnes* is the most commonly isolated organism in periprosthetic infections following shoulder surgery and is a major cause of shoulder prosthesis failure (8, 9).

Current prophylaxis methods such as chlorhexidine gluconate or isopropyl alcohol are ineffective against *C. acnes*. Recent research has documented the greater effectiveness of peroxide-containing compounds in significantly reducing the incidence of *C. acnes* on the skin compared to current standard prophylaxis products. In addition, peroxide products have been shown to have bactericidal activity without developing antibiotic resistance. As the prevalence of shoulder joint prostheses continues to increase, understanding *C. acnes* and preventing infections caused by it is critical (10). However, there is no consensus regarding the recommended compound.

Therefore, we aimed to investigate and compare the effectiveness of the currently available antibacterial substances in shoulder surgery. We hypothesized that peroxide-containing preparations perform better than alcohol-based ones in reducing shoulder specific bacterial flora before shoulder surgery.

## Materials and methods

We have reported this network meta-analysis and systematic review according to the Preferred Reporting Items for Systematic Reviews and Meta-Analyses (PRISMA) Statement 2021 (Table 1). Our protocol was registered on PROSPERO under the registration number. Contrary to what is stated in the protocol, we did not have enough data to investigate dermis swabs and joint culture.

**Table 1:** Basic characteristics of included studies.

Study	Study site	Patients, *n* (male %)	Age, years*	Indication	Intervention	Control	Outcomes – skin/dermis/joint	Germ count
Grewal *et al.* (20)	USA	60 (48)	71.1 ± 7.1 vs 73.4 ± 9.8	Primary arthroplasty	HPO	Standard skin preparation	Culture samples	Positive culture or not
Hancock *et al.* (23)	New Zealand	22 (100)	Adults	Non-surgery, healthy volunteers	BPO	2% CHG and alcohol	Before skin preparation, two swabs were taken from each shoulder region	Positive culture
Hsu *et al.* (22)	USA	50 (100)	Adults	Shoulder arthroplasty	10% BPO soap (BPO group)	4% CHG solution (CHG group)	Skin surface swab, dermal edge swab	Positive culture, SpCuV
Kolakowski *et al.* (25)	USA	80 (46)	Mean: 51	Primary or revision shoulder surgery	5% BPO	4% CHG solution (CHG group)	Skin swab at the anterior, lateral, and posterior arthroscopic portal sites and the axilla	Positive culture
Scheer *et al.* (26)	Sweden	40 (60)	20–66	Non-surgery, healthy volunteers	5% BPO	4% CHG solution (CHG group)	Before and after topical treatment, after surgical skin preparation and sterile draping	Positive culture
Vendela *et al.* (24)	Sweden	100 (63)	Mean: 65/63	Primary elective open shoulder surgery	5% BPO	CHG	After surgical skin preparation, 1 in dermis, and finally after the skin was sutured. Before skin incision, 5 punch biopsies	Positive culture
Stull *et al.* (21)	USA	70 (100)	54.2 ± 13.4 vs 50.1 ± 13.2	Shoulder arthroscopy	Additional with 3% HPO	Standard skin preparation	3 mm punch biopsy was obtained from the posterior arthroscopic portal site of all patients	Positive culture
Symonds *et al.* (27)	Australia	101 (60)	68.1 ± 6.9; 70 ± 5.9; 67.9 ± 7.6	Total shoulder arthroplasty	BPO/BPO + C	Phisohex solution	Swab1-skin, Swab2-skin, Swab3-dermis, Swab4-joint, Swab5-dermis, Swab6-surgical. Trolley	Positive culture
Unterfrauner *et al.* (28)	Switzerland	60 (45)	59	Open-shoulder surgery	5% BPO/2% MN	Standard skin preparation	Swab1-skin, Swab2-skin, Swab3-subcutaneous Swab4-capsule	Positive culture
Van Diek *et al.* (29)	Netherlands	29	57.2 (8.6)	Healthy participants	5% BPO	Placebo	Skin	Positive culture

* Values presented as the mean with standard deviation, or median with range (minimum and maximum).

BPO, benzoil peroxide; BPO + C, benzoil peroxide plus clindamycin; HPO, hydrogen peroxide; MN, miconasole nitrate; CHG, chlorhexidine gluconate; SpCuV, specimen Cutibacterium value.

### Search strategy

A systematic search was conducted in MEDLINE (via PubMed), Embase, and the Cochrane Central Register of Controlled Trials (CENTRAL) for studies published until October 2022, and selection was updated on January 20, 2025. The following search terms were used in all databases: ‘shoulder’ AND (*Cutibacterium* OR *Cutibacter* OR Propioni* OR ‘*C. acnes*’ OR ‘*P. acnes*’ OR acnes) AND (hydrogen OR hyperol OR HP OR H2O2 OR benzoyl OR benzil OR ‘benzoyl-peroxide’). No restrictions were applied during the search. In addition, the reference lists of the included studies were screened for additional eligible articles.

### Eligibility criteria and selection strategy

The yield of the search was combined with the reference manager software (EndNote X9; Clarivate Analytics, USA). After the automatic and manual removal of duplicate records, two independent authors evaluated all studies’ titles, abstracts, and full texts. First, we simultaneously performed a primary selection based on the title and abstract. Then, we assessed full texts for inclusion. Finally, a third author (GS) resolved disagreements.

We used the PICO framework to assess study eligibility. We included only randomized-controlled trials (RCTs) that investigated patients’ shoulder skin (P) and compared different skin preparations (hydrogen peroxide – H_2_O_2_, 5% benzoyl peroxide – BPO 5%, 5% benzoyl peroxide with clindamycin – BPO 5% CLI, 5% benzoyl peroxide with miconazole-N – BPO 5% CLI MN, 10% benzoyl peroxide – BPO 10%, 3% hexachlorophene – pHisohex, chlorhexidine gluconate – CHG, and control). The outcomes (O) we examined were the reduced *C. acnes* culture in skin swabs, dermis swabs, and joint culture. Our study compared alcohol-based skin preparation with alcohol-based skin preparation supplemented with peroxide solutions. If there was less *C. acnes* outgrowth after skin treatment, this was considered a more favourable outcome. After sampling, the bacteria were cultured in anaerobic conditions for at least 12 days. Abstracts and grey literature (preprints and other non-peer-reviewed material) were excluded from the analysis.

### Data extraction

Two independent review authors (VW and BH) extracted data in duplicate into a standardized data collection form (Microsoft Excel 365, Microsoft Corporation, USA). Disagreements were resolved by a third party (GS). We used a standardized data collection sheet to collect all the necessary data: first author; publication year; study design; the clinical outcome was the skin dermis, and the joint culture after peroxide solution compared with the control group. A third party (GS) resolved discrepancies. The authors of the eligible articles were not contacted for further information.

### Statistical analysis

Primary data extraction and organization was carried out in Microsoft Excel. We performed a frequentist network meta-analysis to compare the effectiveness of different skin preparations in reducing *C. acnes* colonization. This approach allows the comparison of multiple interventions and the integration of direct and indirect evidence across a connected network of interventions. For each pairwise comparison, we calculated risk ratios (RRs) and their 95% confidence intervals (CIs) as the measure of effect size. We used a random-effects approach since we expected considerable between-study heterogeneity. The variance component was estimated using restricted maximum likelihood (REML). Multi-arm studies were included using multi-arm correction methods to ensure accurate variance estimates and avoid double-counting participants. Treatments were ranked using the P-score metric, which quantifies the certainty that an intervention is better than another based on point estimates and standard errors (11, 12, 13). Diagnostics for consistency and reliability included netsplit analysis, direct evidence proportion plots, minimal parallelism plots, and mean path length plots. These methods evaluated the robustness of the effect size estimates by distinguishing between direct and indirect evidence. Due to the limited number of studies, formal tests for publication bias, such as funnel plots, were not performed.

The results were visualized using forest plots (where interventions are compared to a reference, in our case: “none”), league tables (where the upper triangle shows direct pairwise comparisons, while the lower triangle shows the results of network meta-analysis), network graphs (where circles at the vertices represent intervention arms and edges represent direct comparisons), and rank plots (where treatments are ranked based on the aforementioned P-score metric), providing a comprehensive summary of the intervention comparisons. All analyses were performed in the R (version 4.4.1; 2024-06-14) using the {meta} (11, 13, 14) and {qgraph} packages (11, 14, 15, 16).

### Risk of bias (RoB) assessment

The RoB assessment was performed independently by two authors (VW and BH) using the RoB 2 tool for RCTs recommended by the Cochrane Collaboration. Disagreements were resolved by consensus. We used the robvis web app tool to visualize the RoB assessment (17).

### Certainty of evidence

The Grades of Recommendation, Assessment, Development and Evaluation (GRADE) Working Group modality approach was used to assess the certainty of evidence. The certainty of the evidence was independently assessed by two authors (VW and BH), and disagreements were resolved by consensus (Supplementary Table 1 (see section on Supplementary materials given at the end of the article)).

## Results

### Search and selection

**Figure 1:**
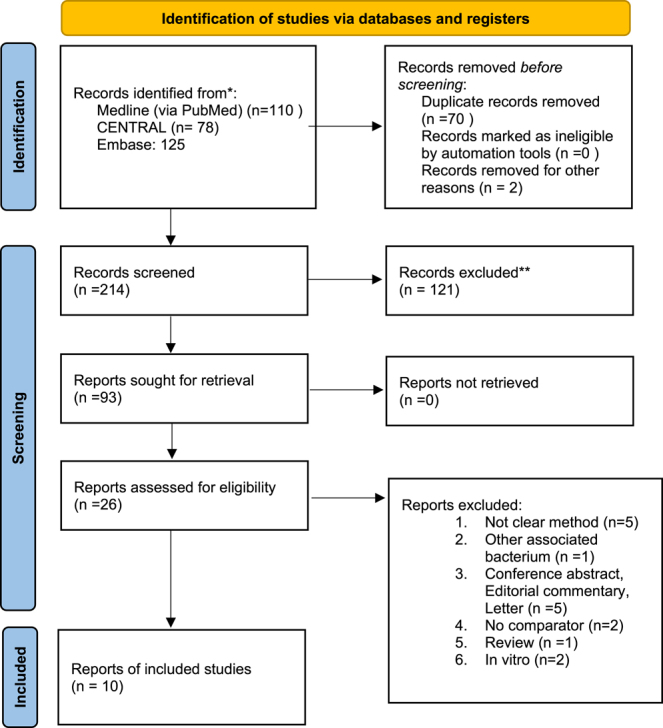
PRISMA 2020 flowchart representing the study selection process.

In total, 214 records were identified through electronic database searches, of which ten publications were included in our network meta-analysis (Fig. 1).

### Basic characteristics of included studies

All included studies were single-centre RCTs. Two of the ten articles compared the H_2_O_2_ with 4% CHG (18, 19). One of them compared the 10% BPO with 4% CHG (20) and three compared 5% BPO with the 4% CHG (21, 22, 23). One article compared the 5% BPO with regular soap (24). One article compared the 5% BPO with phisohex and 5% BPO + clindamycin (25). One article used 5% BPO plus MN (26), and one compared the 5% BPO with placebo (27).

A detailed description of the included studies is shown in Table 2. The network graph of the included publications’ interventions is presented in Supplementary Fig. 1.

**Table 2:** League table. The intervention groups are presented in bold.

V1	V2	V3	V4	V5	V6	V7	V8
**BPO10%**				1.00 (0.41; 2.45)			
1.42 (0.44; 4.57)	**BPO5%**	1.00 (0.23; 4.26)		0.71 (0.33; 1.50)		0.25 (0.08; 0.72)	0.38 (0.11; 1.35)
1.42 (0.22; 9.13)	1.00 (0.23; 4.26)	**BPO5% CLI**					0.38 (0.11; 1.35)
0.44 (0.06; 2.95)	0.31 (0.07; 1.39)	0.31 (0.04; 2.50)	**BPO5% MN**			0.80 (0.28; 2.30)	
1.00 (0.41; 2.45)	0.71 (0.33; 1.50)	0.71 (0.14; 3.62)	2.29 (0.42; 12.38)	**CHG**	0.75 (0.14; 3.98)		
0.75 (0.11; 4.99)	0.53 (0.08; 3.30)	0.53 (0.05; 5.47)	1.72 (0.16; 18.44)	0.75 (0.14; 3.98)	**H2O2**		
0.35 (0.07; 1.71)	0.25 (0.08; 0.72)	0.25 (0.04; 1.50)	0.80 (0.28; 2.30)	0.35 (0.09; 1.30)	0.47 (0.06; 3.89)	**None**	
0.54 (0.10; 3.02)	0.38 (0.11; 1.35)	0.38 (0.11; 1.35)	1.23 (0.17; 8.84)	0.54 (0.12; 2.35)	0.72 (0.08; 6.64)	1.54 (0.29; 8.13)	**Phisohex**

Our network meta-analysis compared the *C. acnes* reduction potential of different interventions. We considered the reducing effect of skin flora in our analysis. We did not have enough data on the dermis and articular bacterial flora to perform the analysis.

### Effectiveness of different interventions to eradicate *C. acnes* on the skin

**Figure 2:**
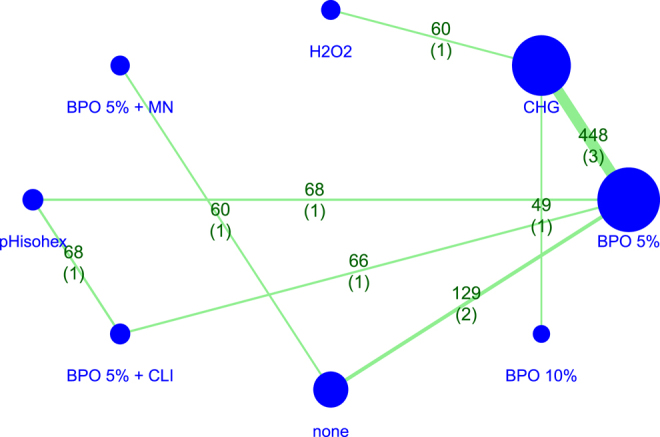
The network graph shows direct comparisons of different interventions.

**Figure 3:**
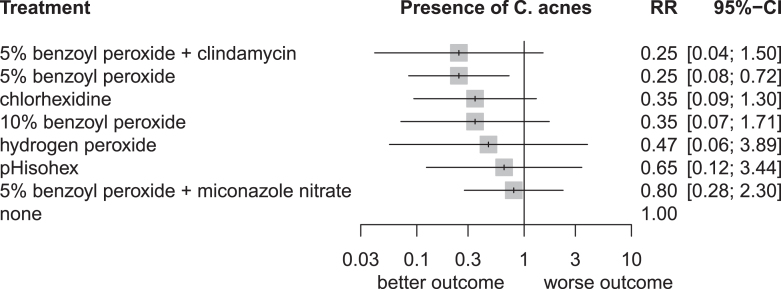
Forest plots representing the odds of the positive *C. acnes* culture on skin.

In our network meta-analysis, we compared seven different interventions from ten different articles. In total, we examined 948 intervention events. Figure 2 shows the network graph with the total number of interventions, the number of comparisons, and the number of comparative studies. The forest plot (Fig. 3) shows that 5% benzyl peroxide was the only intervention with a significant difference compared to the control group (RR = 0.25, CI: 0.08–0.72). 5% BPO, in addition to clindamycin, reduced the *Cutibacterium* flora of the skin on the same level. However, the difference was non-significant compared to the control (RR = 0.25, CI: 0.04–1.50). 5% BPO with MN showed the smallest difference compared to the control group (RR = 0.80, CI: 0.28–2.30).

**Figure 4:**
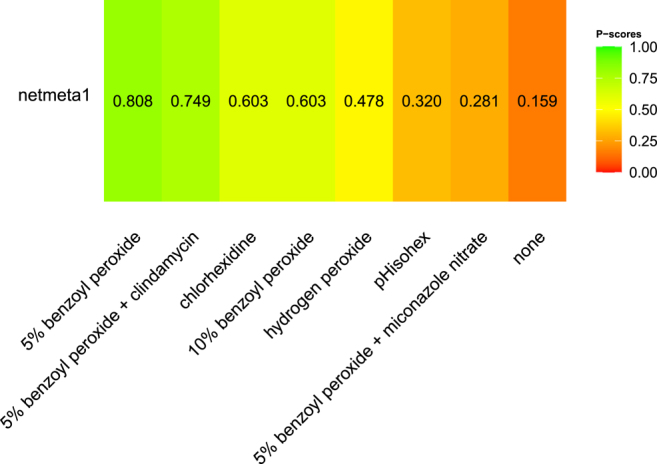
Rank plots representing the most effective material to reduce the *C. acnes* culture on skin.

Based on the rank plot (Fig. 4), 5% BPO (P score: 0.808) proved to be the most effective treatment, followed by BPO 5% plus CLI (P score: 0.749).

Based on the league table (Table 2), we could not find any other significant comparison.

### Effectiveness of different interventions to eradicate *C. acnes* on the dermis

Four articles reported on dermis samples (18, 20, 25, 26). There was no difference in the form of H_2_O_2_ intervention compared to the control group. When 10% BPO was used as an intervention, it reduced the number of *C. acnes* cultured in the dermis more than CHG as a control. In the article comparing BPO/BPO + CLI/phisohex, the best result for reducing dermis *C. acnes* was BPO applied in non-combination, but the combination with CLI also gave better results than the plain phisohex solution. BPO 5% supplemented with 2% MN reduced *C. acnes* better than no intervention ointment.

### Effectiveness of different interventions to eradicate *C. acnes* in the joint

Three articles dealt with the joint sample (18, 25, 26). There was no difference in the samples taken for H_2_O_2_. In none of the cases did *C acnes* grow out of the joint. When 5% BPO and 5% BPO + CLI were tested, there were fewer cases of *C. acnes* colonization with these substances than with phisohex solution, but no difference between BPO in combination and BPO alone was found.

### Risk of bias assessment and certainty of the evidence

Most of the included articles were judged as low-to-moderate (‘some concerns’) RoB, except one article, which had a high RoB (due to ‘bias in measurement in outcome’). A summary of the RoB assessment can be found in the supplementary material (Supplementary Figs 1 and 2).

The grade of evidence was of moderate or very low quality for each pairwise comparison between interventions.

## Discussion

Although *C. acnes* is often associated with surgical site infection following shoulder surgery, it is largely unaffected by standard preoperative skin disinfectants. Our research has shown that BPO significantly reduces *C. acnes* bacterial counts and that 5% BPO was the best count-reducing treatment for this.

The diagnosis and treatment of *C. acnes* can be particularly challenging. The literature suggests that adding peroxide-containing agents to current prophylaxis methods can significantly reduce the number of *C. acnes* copies compared to standard chlorhexidine preparations (19, 22, 27, 28, 29, 30). However, the results are still inconsistent, and we conducted our study to resolve this inconsistency.

Numerous studies incorporated a 5% BPO concentration. Scheer *et al.* conducted an investigation comparing the application of 5% BPO five times to the standard three-time application of 4% CHG. Their results indicated that using 5% BPO significantly decreased the presence of *C. acnes* after preoperative preparation (22, 31). Van Diek *et al.* showed a significant reduction in the presence of *C. acnes* on the skin of the shoulder after BPO compared to placebo. The BPO application involved treating the entire shoulder and axilla region five times, commencing 48 h before the surgical procedure (27, 31). Kolakowski and colleagues also demonstrated a reduction in colonization units when 5% BPO gel was applied a few days before surgery compared to CHG. They showed that applying BPO three times daily resulted in a significant reduction in *C. acnes* bacterial load on the anterior and posterior portals compared to the daily application of CHG. They used a technique that allowed for the quantification of *C. acnes* in sebaceous glands, suggesting that benzoyl peroxide is better able to penetrate deeper layers than chlorhexidine gluconate, which previous studies have shown to be ineffective in eradicating *C. acnes* from the skin (23, 31). BPO reduced *C. acnes* on the shoulder more effectively than chlorhexidine (23, 29).

In contrast to the above studies, Hancock and colleagues found that a single application at the time of surgery does not reduce *C. acnes*. Their research suggests that multiple applications before the day of surgery are necessary to reduce the burden. This may be because *C. acnes* tends to reside in the pilosebaceous glands (21). Similarly, Hsu *et al.* investigated the use of 10% BPO soap applied before surgery compared to CHG, which showed no reduction in *C. acnes* before standard preparation (20, 31).

The combination of BPO with clindamycin has been shown in several studies in the dermatological literature to reduce the colonisation of *C. acnes* (19, 29, 32). The combination of these agents, by penetrating the deep dermal layer, provides a potent local antimicrobial prophylaxis and inhibits the development of antibiotic resistance. This combination was found to have the same efficacy as BPO 5% alone; however, the effect was not statistically significant.

Dizay *et al.* showed a reduction in deep tissue *C. acnes* bacterial load in patients who received daily BPO and clindamycin cream combination therapy for an average of 2–3 days (25, 29). Topical BPO and benzoyl peroxide with clindamycin has been shown to reduce the bacterial load of *C. acnes* on the skin.

H_2_O_2_ has also been shown to be effective in reducing the copy number of *C. acnes*, as Stull *et al.* showed that the addition of hydrogen peroxide to preoperative surgical skin preparation significantly reduces the culture rate of *C. acnes* in the shoulder in the perioperative period (19, 31).

Another topical agent is miconazole nitrate (MN), a broad-spectrum imidazole derivative that interferes with lipid synthesis and *C. acnes* membrane permeability. In this way, the synergistic MN promotes the penetration of BPO into bacterial cells and ultimately supports improved tolerability of topical combination therapy.

In treating acne vulgaris, BPO and MN cream effectively reduce the superficial *C. acnes* load on the skin (26).

From the above and our overall results, it can be concluded that adding BPO can successfully reduce the copy number of *C. acnes* on the skin before shoulder surgery. Reducing the germ count of *C. acnes* can reduce intraoperative wound infection and postoperative infection. BPO should be considered an adjunctive preoperative modality, given its potential benefits, low risk, and low cost.

### Strengths and limitation

Regarding the strengths of our study, the study utilized a network meta-analysis, which evaluated multiple dermal solutions’ efficacy in reducing *C. acne* flora before shoulder surgeries. This method thoroughly compared various interventions, providing valuable insights into their relative effectiveness. Including RCTs ensured a higher level of evidence in the study. Our study was registered on PROSPERO, ensuring transparency.

On the other hand, our study has several limitations. The finding that only one intervention showed a statistically significant difference compared to the control may indicate a lack of consistent and strong evidence for the efficacy of the evaluated dermal solutions. This could be due to the limited number of eligible RCTs or variations in study design and patient populations. The inability to test for publication bias might introduce the possibility of selective reporting of positive outcomes, potentially skewing the overall conclusions. The observed heterogeneity in the outcomes of the included studies may limit the ability to draw definitive conclusions. Variability in study designs, patient characteristics, and intervention protocols could contribute to this heterogeneity. The focus solely on *C. acnes* eradication from the skin, not from the dermis or the joint, might limit the study’s applicability to the broader context of shoulder surgeries.

### Implication for practice and research

By supplementing alcohol-based skin disinfectants with peroxide-containing agents, we have a good chance of reducing the incidence of septic complications, the most dreaded in orthopaedic surgery.

In addition, further research with a broader range of outcomes and more standardized protocols may be needed to strengthen the evidence in this field. It is recommended that the skin surface, dermis, and intra-articular cultures be investigated, taking into account different time frames. Surgical outcomes should also be evaluated. Finally, the rapid application of scientific results is of utmost importance (33, 34).

## Conclusion

Based on our network meta-analysis, we can conclude that the most effective agent to reduce the colonization of skin surface *C. acnes* is 5% BPO.

## Supplementary materials



## ICMJE Statement of Interest

The authors declare that there is no conflict of interest that could be perceived as prejudicing the impartiality of the work reported.

## Funding Statement

This work did not receive any specific grant from any funding agency in the public, commercial or not-for-profit sector.

## Data availability

The datasets used in this study can be found in the full-text articles included in the systematic review and meta-analysis.
