# Supplementary material for: 5% benzoyl peroxide is the most efficient in reducing the cutibacterium flora of the shoulder skin: a network meta-analysis
Source: EFORT Open Rev. 2025 Jun 30;10(7):543–50. doi: 10.1530/EOR-2024-0160 (PMC12232397; doi:10.1530/EOR-2024-0160)

# *5% Benzoyl Peroxide Is the Most Efficient in Reducing the Cutibacterium Flora of the Shoulder Skin: A Network Meta-Analysis*

## Supplementary Tables

Supplementary table 1. GRADE evidence profile – Comparison: Skin culture reduce

## Supplementary Figures

Supplementary figure 1. RoB 2 –Traffic Light Plot

Supplementary figure 2. RoB 2 – VAS 0-4 weeks, Summary Plot

**Summary of findings:**

## Peroxide compared to alcohol based skin preparaion for reduce C. Acnes culture on skin

**Patient or population:** reduce C. Acnes culture on skin

**Setting:**

**Intervention:** peroxide

**Comparison:** alcohol based skin preparaion

| Outcomes | Anticipated absolute effects* (95% CI) | | Relative effect (95% CI) | № of participants (studies) | Certainty of the evidence (GRADE) | Comments |
|---|---|---|---|---|---|---|
| | **Risk with alcohol based skin preparaion** | **Risk with peroxide** | | | | |
| Alcohol based skin prep+Additional 5%BPO | 305 per 1 000 | **0 per 1 000** (24 to 220) | **RR 0.00** (0.08 to 0.72) | 645 (0 RCTs) | ⨁◯◯◯ Very low | |
| Alcohol based skin prep+Additional 10%BPO | 1 000 per 1 000 | **0 per 1 000** (70 to 1 000) | **RR 0.00** (0.07 to 1.71) | 49 (0 RCTs) | ⨁◯◯◯ Very low | |
| Alcohol based skin prep+Additional 5%BPO+Cli | 400 per 1 000 | **0 per 1 000** (56 to 600) | **RR 0.00** (0.14 to 1.50) | 68 (0 RCTs) | ⨁◯◯◯ Very low | |
| Alcohol based skin prep+Additional 5%BPO+MN | 500 per 1 000 | **0 per 1 000** (140 to 1 000) | **RR 0.00** (0.28 to 2.30) | 60 (0 RCTs) | ⨁⨁◯◯ Low | |
| New outcomeAlcohol based skin prep+Additional 3% H2O2 | 160 per 1 000 | **0 per 1 000** (10 to 622) | **RR 0.00** (0.06 to 3.89) | 200 (0 RCTs) | ⨁⨁◯◯ Low | |

***The risk in the intervention group** (and its 95% confidence interval) is based on the assumed risk in the comparison group and the **relative effect** of the intervention (and its 95% CI).

**CI:** confidence interval; **RR:** risk ratio

**GRADE Working Group grades of evidence**
**High certainty:** we are very confident that the true effect lies close to that of the estimate of the effect.
**Moderate certainty:** we are moderately confident in the effect estimate: the true effect is likely to be close to the estimate of the effect, but there is a possibility that it is substantially different.
**Low certainty:** our confidence in the effect estimate is limited: the true effect may be substantially different from the estimate of the effect.
**Very low certainty:** we have very little confidence in the effect estimate: the true effect is likely to be substantially different from the estimate of effect.

**Supplementary Figure 1.** Risk of bias assessment in randomized control trial with RoB 2 Traffic Light Plot

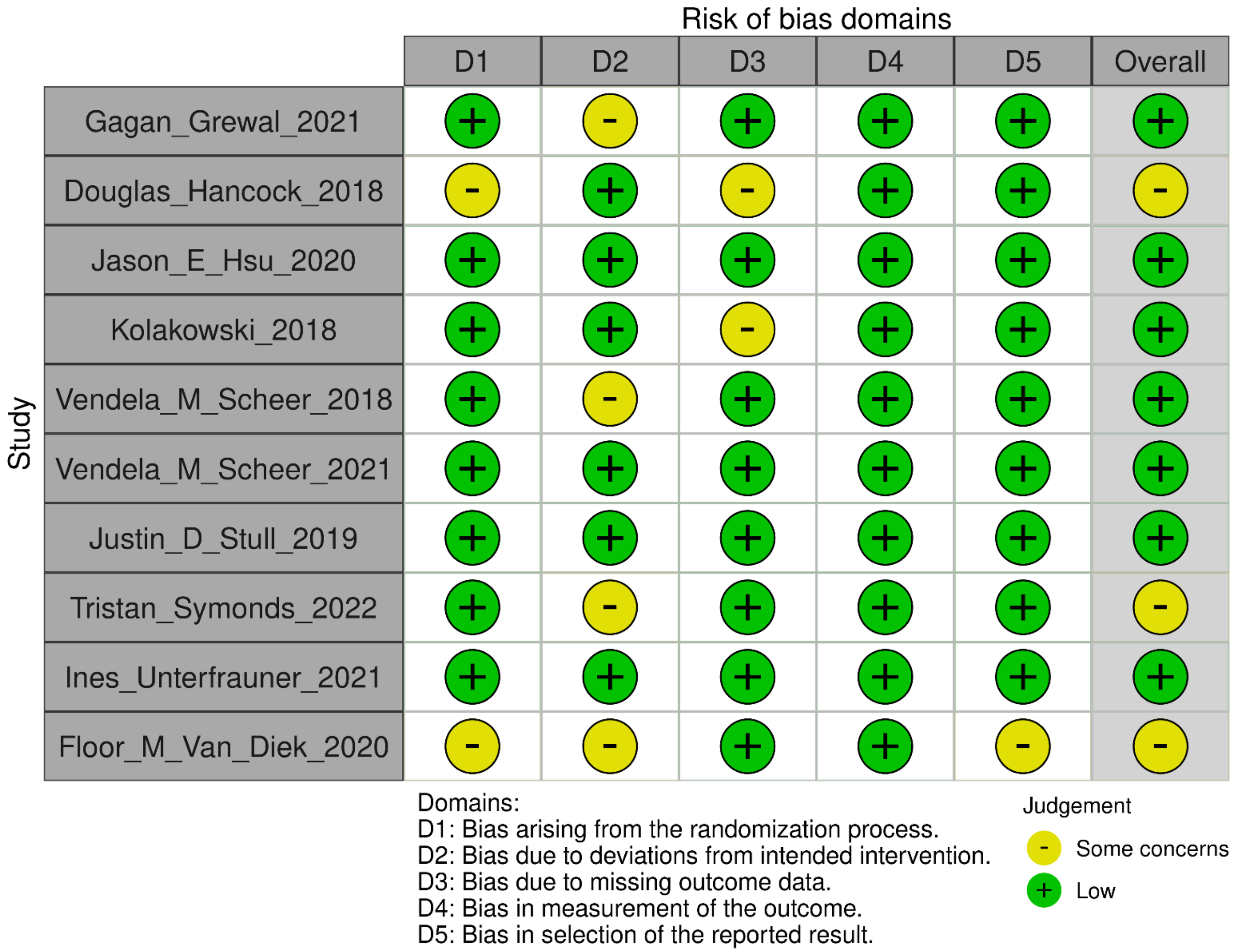

**Supplementary Figure 2.** Risk of bias assessment in randomized control trial with RoB 2 Summary Plot

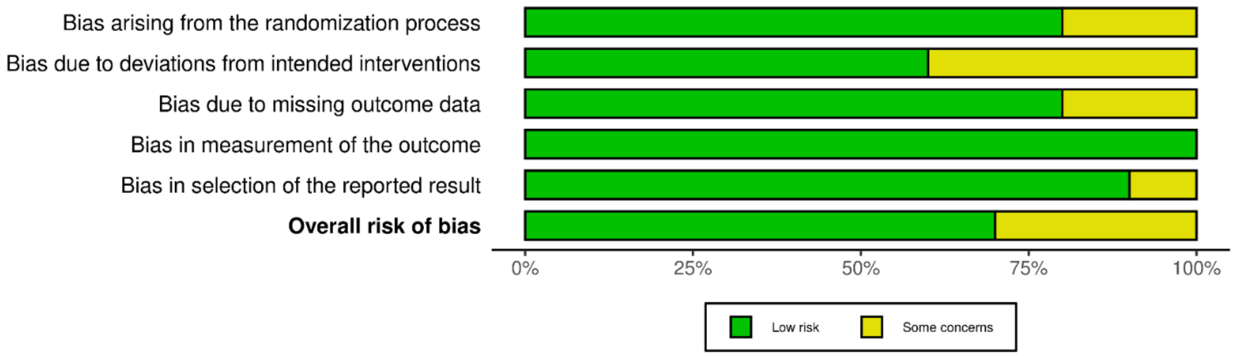

Supplement: Supplementary file 1 [file supplementary_materials.pdf]
